# Applications of Propargyl Esters of Amino Acids in Solution-Phase Peptide Synthesis

**DOI:** 10.1155/2011/854952

**Published:** 2011-06-16

**Authors:** Ramesh Ramapanicker, Rohit Gupta, Rajendran Megha, Srinivasan Chandrasekaran

**Affiliations:** Department of Organic Chemistry, Indian Institute of Science, Bangalore 560012, India

## Abstract

Propargyl esters are employed as effective protecting groups for the carboxyl group during solution-phase peptide synthesis. The propargyl ester groups can be introduced onto free amino acids by treating them with propargyl alcohol saturated with HCl. The reaction between propargyl groups and tetrathiomolybdate is exploited to deblock the propargyl esters. The removal of the propargyl group with the neutral reagent tetrathiomolybdate ensures that most of the other protecting groups used in peptide synthesis are untouched. Both acid labile and base labile protecting groups can be removed in the presence of a propargyl ester. Amino acids protected as propargyl esters are employed to synthesize di- to tetrapeptides in solution-phase demonstrating the possible synthetic utilities of the methodology. The methodology described here could be a valuable addition to currently available strategies for peptide synthesis.

## 1. Introduction

Several methods are available for the protection of the carboxyl group of amino acids during peptide synthesis [1]. However, with the advent of combinatorial chemistry and with medicinal chemistry developing into a separate branch of science, the use of amino acids having multiple functionalities and which are different from the natural amino acids has become very common. This has also brought up the requirement of additional protecting groups, which are orthogonal to those being used. The *α*-carboxyl groups of amino acids are commonly protected as methyl, benzyl, *t*-butyl, allyl and fluorenylmethyl esters [1]. A useful new protecting group should not only be orthogonal to at least a few of the above esters but also should be complementary to the amino and hydroxy protecting groups used in peptide synthesis. 

Various reports from our laboratory have demonstrated the curious reactivity of propargyl systems with tetrathiomolybdate [2]. It has been shown that propargyl ethers [3] and propargyl esters [4] undergo cleavage in the presence of benzyltriethylammonium tetrathiomolybdate (**1**) to give alcohols and acids respectively. Propargyloxycarbonyl (Poc) group, which can be deprotected with tetrathiomolybdate (**1**), has been used as an efficient protecting group for amines [5, 6] and alcohols [7–9], and its applications in peptide synthesis have been established [6, 9]. Herein, we report a systematic study demonstrating the utility of propargyl esters as a protecting group for carboxyl groups in solution-phase peptide synthesis. 

## 2. Results and Discussion

**Scheme 1:**
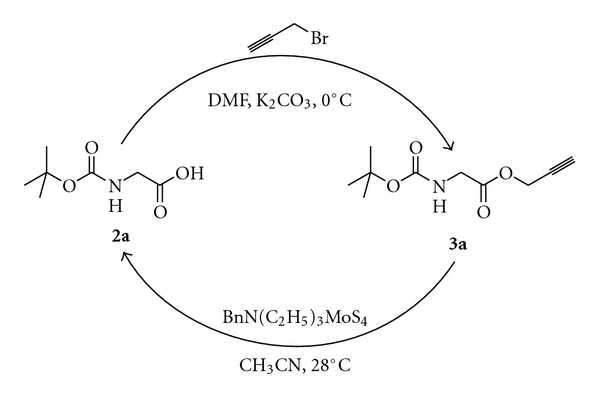
Preparation of the propargyl ester of Boc-Gly-OH **(2a)** and the effective deprotection of the propargyl ester with tetrathiomolybdate.

**Scheme 2:**
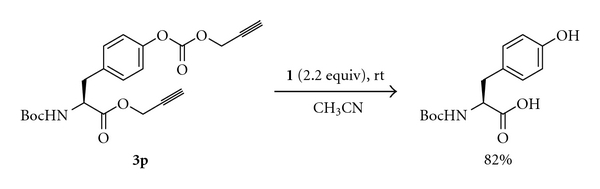
Simultaneous deprotection of a propargyl ester and a propargyloxycarbonyl group.

Propargyl (Prp) ester derivatives of a number of *N*-protected amino acids were prepared by treating them with propargyl bromide (DMF, K_2_CO_3_, 0°C). The propargyl esters were obtained in excellent yields under the conditions employed (Scheme 1, Table 1). As expected these propargyl esters could be deprotected very effectively using 1 equiv of tetrathiomolybdate (**1**, CH_3_CN, 28°C, 2 h). The reactions were very clean, and the products were obtained in high yields (Scheme 1, Table 1). The side chain carboxyl groups of aspartic acid and glutamic acids could also be protected as propargyl esters and could efficiently be deprotected (entries 9 and 12 in Table 1). It is notable that the propargyl ester of 2-aminoisobutyric acid, which is much hindered compared to the other examples studied, could be deprotected in good yield (entry 8, Table 1). Base sensitive Fmoc group in Fmoc-Ala-OPrp (**3m**) was unaffected by the treatment with **1**, whereas the propargyl ester was deblocked to give **2m** (entry 13, Table 1). The primary amide group of asparagine was also not affected by the conditions used for the deprotection of the propargyl ester (entry 14, Table 1). The deprotection of propargyl ester with **1** leaves a *t*-butyl, an allyl or a methyl ester unaffected (entries 9–12, Table 1). Tetrathiomolybdate (**1**) is used for the deprotection of propargyl carbonates [7–9]. However, a benzyl carbonate as in Boc-Tyr(Cbz)-OPrp (**3o**) is unaffected on treatment with **1**, which removes the propargyl ester selectively (entry 15, Table 1). It has to be noted that Boc-Tyr(Poc)-OPrp (**3p**), which can be prepared from Boc-Tyr-OH on treatment with PocCl (2 equiv, Et_3_N, CH_2_Cl_2_, −70°C to rt, 3 h) [10], reacts with 2 equiv of **1** resulting in the deprotection of both the propargyloxycarbonyl group and the propargyl ester (Scheme 2). The reaction of Boc-Ile-OPrp (**3d**) with tetrathiomolybdate (**1**) yielded **2d** in 85% yield. The reaction did not produce any other diastereomer of Boc-Ile-OH (**2d**) suggesting that the deprotection of propargyl esters with **1** does not result in the racemization of amino acids, as evident from the ^1^H NMR spectrum of the compound. These results suggest that the propargyl esters of amino acids can be used for the protection of the carboxyl group.

**Scheme 3:**
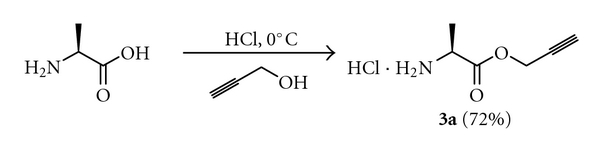
Preparation of the propargyl ester of alanine.

In order for the methodology to be useful in peptide synthesis, it is required that propargyl esters can be prepared from *N*-unprotected amino acids in good yields. Our efforts to esterify alanine with excess propargyl alcohol in the presence of SOCl_2_ were unsuccessful. Similarly, reacting alanine and propargyl alcohol in the presence of catalytic amount of *p*-toluenesulfonic acid in benzene, with azeotropic removal of water, was also unsuccessful. The yields of the ester obtained were very poor, and the reaction mixture turned dark, probably from the polymerization of propargyl alcohol. However, when alanine was treated with propargyl alcohol saturated with HCl, propargyl ester of alanine (**4a**) was obtained in 72% yield (Scheme 3). The procedure was repeated with a number of other amino acids and the corresponding propargyl esters could be isolated in good yields (Table 2). The products were initially obtained as brownish residues, which had to be washed many times with diethyl ether to remove all the impurities. Valine and isoleucine, which are substituted at the *β*-carbon atom, did not react completely. The products were mixtures of the hydrochloride salt of the amino acid and its propargyl ester. The propargyl ester of 2-aminoisobutyric acid (Aib) could not be made even in trace amounts using this procedure. Although substituted at the *β*-carbon atom, threonine reacted with propargyl alcohol and HCl·H-Thr-OPrp (**4h**) could be isolated in 69% yield as a single diastereomer, indicating the absence of racemization at the *α*-carbon under these conditions (entry 8, Table 2), as evident from the ^1^H NMR spectrum of the compound. The procedure could also be used for preparing the propargyl esters (**4i** and** 4j**) of 4- and 3-aminobenzoic acid in very good yields (entry 9 and 10, Table 2).

**Scheme 4:**
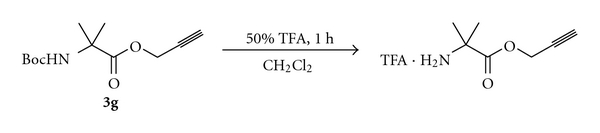
Preparation of the propargyl ester of Aib from Boc-Aib-OPrp.

The treatment of HCl·H-Ala-OPrp (**4a**) with neat TFA or 20% piperidine in DMF did not result in the deprotection of the propargyl ester. Therefore, propargyl esters can be used with *t*-butyl based and Fmoc protecting groups. Although propargyl esters can be cleaved using **1** in the presence of an allyl ester (entries 10 and 11, Table 1), cleavage of allyl esters using Pd(PPh_3_)_4_ and a nucleophile [1] results in the cleavage of propargyl esters (Scheme 4). The results suggest that propargyl esters of amino acids are suitable for solution-phase peptide synthesis, especially when the *α*-amino group is protected as a Boc derivative.

Although propargyl esters of some amino acids could not be prepared directly, they could be prepared from the Boc derivatives of these amino acids (Scheme 1). The cleavage of Boc using TFA in CH_2_Cl_2_ can then provide propargyl esters of such amino acids, which are otherwise difficult to synthesize (Scheme 4). Trifluoroacetic acid salts of the amino propargyl esters thus obtained can directly be used for peptide synthesis. We used this strategy for the preparation of dipeptides from the propargyl esters of Aib, Val, and Ile (Table 3).

**Scheme 5:**
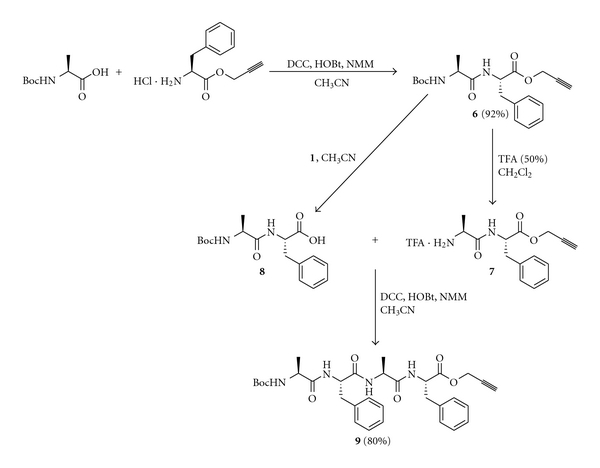
Synthesis of a tetrapeptide using propargyl ester for carboxyl protection.

Finally to demonstrate the usefulness of the methodology, we synthesized a tetrapeptide through a fragment condensation strategy, which employed the deprotection of propargyl ester with tetrathiomolybdate (**1**) as one of the key steps. Boc-Ala-OH was coupled with HCl·H-Phe-OPrp (DCC, HOBt, NMM, CH_3_CN) to get the dipeptide Boc-Ala-Phe-OPrp (**6**) in 92% yield. Treating a fraction of **6** with TFA (50% in CH_2_Cl_2_) gave the free amine TFA·H-Ala-Phe-OPrp (**7**) and treating another fraction of **6** with tetrathiomolybdate (**1**) gave the free acid Boc-Ala-Phe-OH (**8**). The amino component **7** and the carboxyl component **8** were then coupled together (DCC, HOBt, NMM, CH_3_CN) to get the tetrapeptide Boc-Ala-Phe-Ala-Phe-OPrp (**9**) in 80% yield (Scheme 5). 

## 3. Conclusion

In conclusion, we have demonstrated the utility of propargyl ester as an efficient protecting group for the carboxyl function in solution-phase peptide synthesis. The propargyl ester group is deprotected using the neutral reagent benzyltriethylammonium tetrathiomolybdate, which does not react with other commonly used protecting groups. The introduction or deprotection of the propargyl esters did not result in racemization of the amino acids. Propargyl esters are stable to the conditions used for the deprotection of Fmoc and *t*-butyl-based protecting groups. We have shown the application of the methodology by synthesizing a tetrapeptide. 

## 4. Experimental

### 4.1. General Experimental Procedures

Melting points and optical rotation (at 25°C) were recorded on digital instruments. Infrared spectra were recorded using an FT-IR instrument the frequencies are reported in wave number (cm^−1^), and intensities of the peaks are denoted as s (strong), w (weak), and m (medium). ^1^H and ^13^C NMR spectra were recorded on a 300 MHz and 75 MHz spectrometer respectively. Chemical shifts are reported in parts per million downfield from the internal reference, tetramethylsilane. Multiplicity is indicated using the following abbreviations: s (singlet), d (doublet), t (triplet), q (quartet), m (multiplet), dd (double doublet), bs (broad singlet), and bd (broad doublet). Coupling constants are reported wherever it is necessary in Hertz (Hz). Mass spectra were recorded on a High-Resolution Q-TOF electrospray instrument. 

### 4.2. Preparation of Benzyltriethylammonium Tetrathiomolybdate

Ammonium molybdate (10 g) was dissolved in a mixture of ammonium hydroxide (60 mL) and water (20 mL), and the solution was filtered. Hydrogen sulfide was bubbled rapidly at room temperature (28°C) into the solution until it was saturated and the temperature was raised to 60°C, while maintaining a slow stream of hydrogen sulfide. After 60 min, the mixture was cooled to 0°C and kept under refrigeration for 30 min. The granular product thus obtained was isolated by filtration. The crystalline solid was washed with isopropyl alcohol (25 mL × 2), ether (25 mL × 4), and dried under vacuum to get brick red crystals of ammonium tetrathiomolybdate (13·4 g, 92%).

A solution of benzyltriethylammonium chloride (23·31 g, 102·5 mmol) in distilled water (60 mL) was added in portions over 30 min to a well-stirred solution of ammonium tetrathiomolybdate (13 g, 50 mL) in distilled water (60 mL). Rapid stirring was continued for 2 h at room temperature, and the solid that separated was filtered, washed with isopropyl alcohol (40 mL × 2) and ether (40 mL × 4). The brick red powder of benzyltriethylammonium tetrathiomolybdate (**1**) was dried under vacuum and stored in a desiccator (24 g, 80%). Melting point: decomposes at 150°C. 

### 4.3. General Procedure for the Synthesis of Propargyl Esters (**3a-o**) of N-Protected Amino Acids

*N*-protected amino acids (**2a-o**, 5 mmol) were dissolved in anhydrous DMF (10 mL) and the solution was cooled to −10°C. Anhydrous K_2_CO_3_ (5 mmol) was added to the solution and the stirring was continued until a syrupy solution is formed. Propargyl bromide (0.55 mL of 80% solution in toluene, 5 mmol) was added dropwise to the reaction mixture, and the stirring continued at −10°C for 1 h. The reaction mixture was then allowed to attain rt, DMF was removed under vacuum, and the residue was extracted with ethyl acetate (50 mL). The solution of the crude product was taken in a separating funnel and washed with saturated citric acid solution (2 × 25 mL), water (2 × 25 mL), and brine (25 mL), dried over anhydrous Na_2_SO_4_ and concentrated. The crude products were then purified using column chromatography (silica gel, 100–200 mesh) using a solution of ethyl acetate (10–30%) in petroleum ether as eluent.

**Boc-Gly-OPrp (3a)**

White crystalline solid; mp 80°C, FTIR (Neat) 3355 (br), 2131 (w), 1757 (s), 1715 (s); ^1^H NMR (CDCl_3_) *δ* 5.06 (bs, 1H), 4.75 (d, *J* = 2.4 Hz, 2H), 3.96 (d, *J* = 6 Hz, 2H), 2.51 (t, *J* = 2.4 Hz, 1H), 1.45 (s, 9H); ^13^C NMR (CDCl_3_) *δ* 169.7, 155.6, 100.5, 80.1, 75.4, 52.6, 42.3, 28.3; ESMS Calculated for C_10_H_15_NO_4_ + Na: 236.2202, Observed 236.2199.

**Boc-*β*Ala-OPrp (3b)**

Pale yellow oil; FTIR (Neat) 3370 (br), 2129 (w), 1742 (s), 1708 (s); ^1^H NMR (CDCl_3_) *δ* 5.20 (bs, 1H), 4.70 (d, *J* = 2.4 Hz, 2H), 3.40 (q, *J* = 6.3 Hz, 2H), 2.58 (d, *J* = 6.3 Hz, 2H), 2.54 (t, *J* = 2.4 Hz. 1H), 1.43 (s, 9H); ^13^C NMR (CDCl_3_) *δ* 171.4, 155.5, 79.1, 74.9, 51.8, 35.8, 34.2, 28.1; ESMS Calculated for C_11_H_17_NO_4_ + Na: 250.2468, Observed 250.2465.

**Boc-Met-OPrp (3c)**

Pale yellow oil; [*α*]_D_  −35 (c = 1, EtOH); FTIR (Neat) 3357 (br), 2129 (w), 1749 (s), 1714 (s); ^1^H NMR (CDCl_3_) *δ* 5.19 (bd, *J* = 7.2 Hz, 1H), 4.79 (dd, *J *_1_ = 15 Hz, *J *_2_ = 2.4 Hz, 1H), 4.70 (dd, *J *_1_ = 15 Hz, *J *_2_ = 2.4 Hz, 1H), 4.44–4.46 (m, 1H), 2.56 (t, *J* = 7.8 Hz, 1H), 2.09–2.19 (m, 4H), 1.91–2.03 (m, 1H), 1.45 (s, 9H); ^13^C NMR (CDCl_3_) *δ* 171.5, 155.2, 80.1, 75.40, 75.37, 52.7, 31.8, 29.8, 28.2, 15.40, 15.36; ESMS Calculated for C_13_H_21_NO_4_S + Na: 310.3649, Observed 310.3649.

**Boc-Val-OPrp (3d)**

Pale yellow oil; [*α*]_D_  −36 (c = 1, EtOH); FTIR (Neat) 3388 (br), 2129 (w), 1747 (s), 1714 (s); ^1^H NMR (CDCl_3_) *δ* 5.02 (bs, 1H), 4.79 (dd, *J *_1_ = 15.4 Hz, *J *_2_ = 1.8 Hz, 1H), 4.68 (dd, *J *_1_ = 15.3 Hz, *J *_2_ = 1.8 Hz, 1H), 4.24–4.29 (m, 1H), 2.47–2.50 (m, 1H), 2.12–2.23 (m, 1H), 1.45 (s, 9H), 0.98 (d, *J* = 6.9 Hz, 3H), 0.92 (d, *J* = 7.2 Hz, 3H); ^13^C NMR (CDCl_3_) *δ* 171.6, 155.6, 79.9, 77.2, 75.2, 58.4, 52.3, 31.3, 28.3, 18.9, 17.5; ESMS Calculated for C_13_H_21_NO_4_ + Na: 278.2999, Observed 278.3001.

**Boc-Ile-OPrp (3e)**

Pale yellow oil; [*α*]_D_  −31 (c = 1, EtOH); FTIR (Neat) 3385 (br), 2130 (w), 1748 (s), 1714 (s); ^1^H NMR (CDCl_3_) *δ* 5.04 (bd, *J* = 7.8 Hz, 1H), 4.80 (dd, *J *_1_ = 15.4 Hz, *J *_2_ = 2.1 Hz, 1H), 4.67 (dd, *J *_1_ = 15.4 Hz, *J *_2_ = 2.1 Hz, 1H), 4.28–4.33 (m, 1H), 2.49 (t, *J* = 2.1 Hz, 1H), 1.89 (bs, 1H), 1.39–1.49 (m, 10H), 1.11–1.27 (m, 1H), 0.90–0.96 (m, 6H); ^13^C NMR (CDCl_3_) *δ* 171.6, 155.5, 79.9, 77.2, 75.1, 57.8, 52.3, 38.1, 28.3, 25.0, 15.5, 11.5; ESMS Calculated for C_14_H_23_NO_4_ + Na: 292.3265, Observed 292.3269.

**Boc-Lys(Boc)-OPrp (3f)**

Pale yellow oil; [*α*]_D_  −24 (c = 1, EtOH); FTIR (Neat) 3353 (br), 2128 (w), 1748 (s), 1697 (s); ^1^H NMR (CDCl_3_) *δ* 5.17 (bd, *J* = 6.6 Hz, 1H), 4.79 (dd, *J *_1_ = 15.4 Hz, *J *_2_ = 2.1 Hz, 1H), 4.68 (dd, *J *_1_ = 15.4 Hz, *J *_2_ = 2.1 Hz, 1H), 4.28–4.35 (m, 1H), 3.08–3.14 (m, 2H), 2.52 (t, *J* = 2.4 Hz, 1H), 1.63–1.89 (m, 2H), 1.35–1.60 (m, 22H); ^13^C NMR (CDCl_3_) *δ* 172.0, 156.0, 155.4, 79.9, 79.1, 77.1, 75.3, 53.1, 52.5, 39.9, 32.0, 29.5, 28.3, 28.2, 22.3; ESMS Calculated for C_19_H_32_N_2_O_6_ + Na: 407.4570, Observed 407.4567.

**Boc-Phg-OPrp (3g)**

White solid; mp 48°C; [*α*]_D_  +32 (c = 1, EtOH); FTIR (Neat) 3402 (br), 2131 (w), 1748 (s), 1722 (s); ^1^H NMR (CDCl_3_) *δ* 7.32–7.38 (m, 5H), 5.51 (bd, *J* = 7 Hz, 1H), 5.36 (bd, *J* = 7.2 Hz, 1H), 4.75 (dd, *J *_1_ = 16 Hz, *J *_2_ = 1.8 Hz, 1H), 4.67 (dd, *J *_1_ = 15 Hz, *J *_2_ = 1.8 Hz, 1H), 2.45 (t, *J* = 2.1 Hz, 1H), 1.43 (s, 9H); ^13^C NMR (CDCl_3_) *δ* 170.4, 154.7, 136.3, 128.9, 128.6, 127.2, 80.3, 76.8, 75.4, 57.5, 53.0, 28.2; ESMS Calculated for C_16_H_19_NO_4_ + Na: 312.3162, Observed 312.3158.

**Boc-Aib-OPrp (3h)**

Pale yellow oil; FTIR (Neat) 3377 (br), 2130 (w), 1746 (s), 1713 (s); ^1^H NMR (CDCl_3_) *δ* 5.09 (bs, 1H), 4.72 (d, *J* = 2.7 Hz, 2H), 2.47 (t, *J* = 2.7 Hz, 1H), 1.51 (s, 6H), 1.44 (s, 6H); ^13^C NMR (CDCl_3_) *δ* 173.9, 154.5, 79.8, 75.0, 56.0, 52.7, 28.2, 25.3; ESMS Calculated for C_12_H_19_NO_4_ + Na: 264.2734, Observed 264.2735.

**Cbz-Asp(OPrp)-O^*t*^Bu (3i)**

Colorless oil; [*α*]_ D _ +83 (c = 1, EtOH); FTIR (Neat) 3293 (br), 2129 (w), 1738 (s), 1732 (s), 1728 (s); ^1^H NMR (CDCl_3_) *δ* 7.32–7.36 (m, 5H), 5.76 (bd, *J* = 7.5 Hz, 1H), 5.11 (s, 2H), 4.50–4.67 (m, 3H), 3.01(dd, *J *_1_ = 17 Hz, *J *_2_ = 4.5 Hz, 1H), 2.86 (dd, *J *_1_ = 17 Hz, *J *_2_ = 4.5 Hz, 1H), 1.45 (s, 9H); ^13^C NMR (CDCl_3_) *δ* 169.9, 169.2, 155.8, 136.1, 128.4, 128.04, 127.96, 82.6, 75.2, 66.9, 52.2, 50.7, 36.6, 27.7; ESMS Calculated for C_19_H_23_NO_6_ + Na: 384.1423, Observed 384.1426.

**Cbz-Asp(OAllyl)-OPrp (3j)**

White solid; mp 50°C; [*α*]_D_  −11 (c = 1, EtOH); FTIR (Neat) 3365 (br), 2130 (w), 1735 (s); ^1^H NMR (CDCl_3_) *δ* 7.33–7.36 (m, 5H), 5.79–5.95 (m, 2H), 5.22–5.34 (m, 2H), 5.13 (s, 2H), 4.68–4.75 (m, 3H), 4.57–4.60 (m,2H), 3.09 (dd, *J *_1_ = 17.1 Hz, *J *_2_ = 4.8 Hz, 1H), 2.90 (dd, *J *_1_ = 17.1 Hz, *J *_2_ = 4.5 Hz, 1H), 2.50 (t, *J* = 2.4 Hz, 1H); ^13^C NMR (CDCl_3_) *δ* 170.3, 169.9, 155.8, 135.9, 131.5, 128.5, 128.2, 128.0, 118.8, 76.8, 75.5, 67.2, 65.8, 53.2, 50.3, 36.5; ESMS Calculated for C_18_H_19_NO_6_ + Na: 368.1110, Observed 368.1106.

**Boc-Asp(OAllyl)-OPrp (3k)**

Pale yellow oil; [*α*]_D_2009−26 (c = 1, EtOH); FTIR (Neat) 3382 (s), 2130 (w), 1737 (s); ^1^H NMR (CDCl_3_) *δ* 5.82–5.97 (m, 1H), 5.52 (d, *J* = 8.1 Hz, 1H), 5.24–5.36 (m, 2H), 4.74–4.75 (m, 3H), 4.59–4.62 (m, 3H), 3.06 (dd, *J *_1_ = 17.4 Hz, *J *_2_ = 4.8 Hz, 1H), 2.88 (dd, *J *_1_ = 17.1 Hz, *J *_2_ = 4.5 Hz, 1H), 2.50 (t, *J* = 2.7 Hz, 1H), 1.45 (s, 9H); ^13^C NMR (CDCl_3_) *δ* 170.4, 170.3, 155.3, 131.6, 118.7, 80.2, 76.6, 75.4, 65.7, 53.1, 49.9, 36.6, 28.2; ESMS Calculated for C_15_H_21_NO_6_ + Na: 334.1267, Observed 334.1249.

**Boc-Asp(OPrp)-OMe (3l)**

Pale yellow oil; [*α*]_D_ −5 (c = 1, EtOH); FTIR (Neat) 3383 (br), 2131 (w), 1746 (s), 1717 (s); ^1^H NMR (CDCl_3_) *δ* 5.55 (bd, *J* = 2.1 Hz, 1H), 4.68–4.71 (m, 2H), 4.57–4.65 (m, 1H), 3.76 (s, 3H), 2.85–3.07 (m, 2H), 2.52–2.55 (m, 1H), 1.45 (s, 9H); ^13^C NMR (CDCl_3_) *δ* 171.2, 170.0, 155.1, 80.0, 75.2, 52.6, 52.2, 51.8, 49.7, 36.5, 28.1; ESMS Calculated for C_13_H_19_NO_6_ + Na: 308.1110, Observed 308.1110.

**Fmoc-Ala-OPrp (3m)**

White solid; mp 88°C; [*α*]_D_  −25 (c = 1, EtOH); FTIR (Neat) 3401 (br), 2130 (w), 1747 (s), 1724 (s); ^1^H NMR (CDCl_3_) *δ* 7.66–7.69 (m, 2H), 7.52–7.56 (m, 2H), 7.21–7.34 (m, 4H), 5.73 (bd, *J* = 7.5 Hz, 1H), 4.58–4.70 (m, 2H), 4.31–4.42 (m, 3H), 4.13–4.17 (m, 1H), 2.44 (t, *J* = 2.4 Hz, 1H), 1.37 (d, *J* = 6.6 Hz, 3H); ^13^C NMR (CDCl_3_) *δ* 172.1, 155.5, 143.4, 140.9, 127.4, 126.7, 124.8, 119.7, 76.8, 75.4, 66.6, 52.5, 49.2, 46.7, 17.8; ESMS Calculated for C_21_H_19_NO_4_ + Na: 372.3697, Observed 372.3701.

**Cbz-Asn-OPrp (3n)**

White solid; mp 123°C; [*α*]_D_  −21 (c = 1, EtOH); FTIR (Neat) 3292 (br), 2130 (w), 1715 (s); ^1^H NMR (CDCl_3_) *δ* 7.30–7.42 (m, 5H), 5.81 (bd, *J* = 6.6 Hz, 1H), 5.14 (s, 2H), 4.80 (d, *J* = 2.1 Hz, 2H), 4.59–4.65 (m, 1H), 3.05 (dd, *J *_1_ = 17 Hz, *J *_2_ = 5 Hz, 1H), 2.95 (dd, *J *_1_ = 17 Hz, *J*_2_ = 5 Hz, 1H), 2.55 (t, *J* = 2.4 Hz, 1H); ^13^C NMR (CDCl_3_) *δ* 168.0, 155.4, 135.6, 128.6, 128.4, 128.1, 115.7, 76.3, 76.1, 67.6, 53.9, 50.5, 21.6; ESMS Calculated for C_15_H_16_N_2_O_5_ + Na: 327.2877, Observed 327.2880.

**Boc-Tyr(Cbz)-OPrp (3o)**

White solid; mp 60°C; [*α*]_D_  −8 (c = 1, EtOH); FTIR (Neat) 3292 (br), 2130 (w), 1760 (s), 1715 (s); ^1^H NMR (CDCl_3_) *δ* 7.35–7.46 (m, 5H), 7.10–7.19 (m, 4H), 5.26 (s, 2H), 4.98 (bd, *J* = 7.5 Hz, 1H), 4.59–4.80 (m, 3H), 3.04–3.18 (m, 2H), 2.51 (t, *J* = 2.4 Hz, 1H), 1.42 (s, 9H); ^13^C NMR (CDCl_3_) *δ* 170.9, 155.0, 153.5, 150.3, 134.8, 133.6, 130.4, 128.7, 128.5, 121.1, 80.1, 75.5, 70.3, 54.2, 52.6, 37.4, 28.2; ESMS Calculated for C_25_H_27_NO_7_ + Na: 476.1686, Observed 476.1684.

**Boc-Tyr(Poc)-OPrp (3p)**

White solid; mp 65°C; [*α*]_D_  −9 (c = 1, EtOH); FTIR (Neat) 3290 (br), 2130 (w), 1756 (s), 1710 (s); ^1^H NMR (CDCl_3_) *δ* 7.19 (d, *J* = 8.7 Hz, 2H), 7.13 (d, *J* = 8.7 Hz, 2H), 4.98 (bd, *J* = 8 Hz, 1H), 4.84 (d, *J* = 2.4 Hz, 2H), 4.64–4.80 (m, 3H), 3.09–3.14 (m, 2H), 2.59 (t, *J* = 2.4 Hz, 1H), 2.52 (t, *J* = 2.4 Hz, 1H), 1.42 (s, 9H); ^13^C NMR (CDCl_3_) *δ* 171.3, 155.3, 153.3, 150.5, 134.2, 130.8, 121.3, 80.5, 76.6, 76.3, 75.9, 56.2, 54.6, 53.0, 37.8, 28.6; ESMS Calculated for C_21_H_23_NO_7_ + Na: 424.3996, Observed 424.3400.

### 4.4. General Procedure for the Deprotection of Propargyl Esters Using Benzyltriethylammonium Tetrathiomolybdate (** 1**)

To a solution of the propargyl esters (**3a-o**, 1 mmol) in acetonitrile (5 mL), benzyltriethylammonium tetrathiomolybdate (**1**, 1·1 mmol, 0·67 g) was added at rt (28°C) and the reaction mixture was stirred for 2 h. Acetonitrile was removed under vacuum, and the residue was extracted with a mixture of ethyl acetate and chloroform (9 : 1). The crude products were purified by column chromatography (silica gel, 100–200 mesh) eluting with a solution of ethyl acetate in petroleum ether or methanol in chloroform. The simultaneous deprotection of the propargyloxycarbonyl group and the propargyl ester in **3p** was carried out the same way using 2.1 equiv of **1**. 

### 4.5. General Procedure for the Synthesis of Propargyl Esters (**4a-j**) of Amino Acids

Dry HCl was bubbled through propargyl alcohol (20 mL) at 0°C for 1 h. The amino acid (5 mmol) is added to the saturated solution of HCl in propargyl alcohol at 0°C, and the stirring was continued for 12 h at rt (28°C). Propargyl alcohol is removed under vacuum and the residue is washed with anhydrous diethyl ether (25 mL × 10), dried under vacuum, and stored in a desiccator.

**HCl·H-Ala-OPrp (4a)**

White solid; mp 88°C; [*α*]_D_  +32 (c = 1, EtOH); FTIR (KBr) 3292 (br), 2125 (w), 1741 (s); ^1^H NMR (D_2_O) *δ* 4.74 (d, *J* = 2.4 Hz, 2H), 4.11 (q, *J* = 7.5 Hz, 1H), 2.85 (t, *J* = 2.4 Hz, 1H), 1.44 (d, *J* = 7.5 Hz, 3H); ^13^C NMR (D_2_O) *δ* 170.8, 77.7, 54.9, 49.6, 15.8; ESMS Calculated for C_6_H_9_NO_2_ + H: 128.0633, Observed 128.0701.

**HCl·H-Gly-OPrp (4b)**

White solid; mp 158°C; FTIR (KBr) 3440 (br), 2129 (w), 1763 (s); ^1^H NMR (D_2_O) *δ* 4.74 (d, *J* = 2.4 Hz, 2H), 3.84 (s, 2H), 2.83 (t, *J* = 2.4 Hz, 1H); ^13^C NMR (D_2_O) *δ* 168.3, 101.4, 77.6, 54.7, 40.9; ESMS Calculated for C_5_H_7_NO_2_ + H: 114.0555, Observed 114.0564.

**HCl·H-Leu-OPrp (4c)**

White solid; mp 125°C; [*α*]_D_  −19 (c = 1, EtOH); FTIR (KBr) 3442 (br), 1952 (w), 1749 (s); ^1^H NMR (D_2_O) *δ* 4.76 (dd, *J *_1_ = 2.5 Hz, *J *_2_ = 1.2 Hz, 2H), 4.07 (t, *J* = 7.2 Hz, 1H), 2.87 (t, *J* = 2.5 Hz, 1H), 1.59–1.79 (m, 3H), 0.85 (d, *J* = 6.3 Hz, 3H), 0.84 (d, *J* = 6.3 Hz, 3H); ^13^C NMR (D_2_O) *δ* 170.9, 77.8, 77.6, 54.9, 52.2, 39.5, 24.8, 22.3, 21.9; ESMS Calculated for C_9_H_15_NO_2_ + H: 170.1182, Observed 170.1179.

**HCl·H-Pro-OPrp (4d)**

White solid; mp 52°C; [*α*]_D_  −76 (c = 1, EtOH); FTIR (KBr) 3410 (br), 2123 (w), 1751 (s); ^1^H NMR (D_2_O) *δ* 4.77 (d, *J* = 2.1 Hz, 2H), 4.42 (dd, *J *_1_ = 8.6 Hz, *J *_2_ = 7.2 Hz, 1H), 3.25–3.39 (m, 2H), 2.88 (t, *J* = 2.4 Hz, 1H), 2.29–2.41 (m, 1H), 1.90–2.14 (m, 3H); ^13^C NMR (D_2_O) *δ* 170.0, 77.8, 77.6, 60.2, 55.2, 47.2, 28.9, 24.1; ESMS Calculated for C_8_H_11_NO_2_ + H: 154.0790, Observed 154.0860.

**HCl·H-Ser-OPrp (4e)**

Pale yellow oil; [*α*]_D_  −40 (c = 1, EtOH); FTIR (Neat) 3434 (br), 2129 (w), 1751 (s); ^1^H NMR (D_2_O) *δ* 4.76–4.77 (m, 2H), 4.18–4.21 (m, 1H), 4.00 (ddd, *J *_1_ = 13 Hz, *J *_2_ = 4.5 Hz, *J *_3_ = 1.8 Hz, 1H), 3.83–3.92 (m, 1H), 2.85 (dd, *J *_1_ = 4.6 Hz, *J *_2_ = 2.7 Hz, 1H); ^13^C NMR (D_2_O) *δ* 168.6, 77.7, 77.5, 59.9, 55.5, 55.1; ESMS Calculated for C_6_H_9_NO_3_ + Na: 166.0480, Observed 166.0491.

**HCl·H-Phe-OPrp (4f)**

White solid; mp 154°C; [*α*]_D_  −40 (c = 1, EtOH); FTIR (KBr) 3288 (br), 2127 (w), 1743 (s); ^1^H NMR (CDCl_3_) *δ* 7.11–7.24 (m, 5H), 4.63 (d, *J* = 2.1 Hz, 2H), 3.68 (dd, *J *_1_ = 7.8 Hz, *J *_2_ = 5.1 Hz, 1H), 3.02 (dd, *J *_1_ = 13.5 Hz, *J *_2 _= 5.1 Hz, 1 H), 2.81 (dd, *J *_1_ = 13.5 Hz, *J *_2_ = 7.8 Hz, 1H), 2.43 (dd, *J *_1_ = 2.7 Hz, *J *_2_ = 1.8 Hz, 1H), 1.49 (bs, 2H); ^13^C NMR (CDCl_3_) *δ* 174.1, 136.7, 129.2, 128.4, 126.7, 77.2, 75.1, 55.5, 52.2, 40.6; ESMS Calculated for C_12_H_13_NO_2_ + H: 204.1030, Observed 204.1024.

**HCl·H-Glu(OPrp)-OPrp (4g)**

White solid; mp 119°C; [*α*]_D_ +50 (c = 1, EtOH); FTIR (KBr) 3435 (br), 2132 (w), 1740 (s); ^1^H NMR (D_2_O) *δ* 4.71–4.72 (m, 2H), 4.58–4.60 (m, 2H), 4.04–4.12 (m, 1H), 2.83 (dd, *J *_1_ = 5.1 Hz, *J *_2_ = 2.7 Hz, 1H), 2.76 (dd, *J *_1_ = 5.1 Hz, *J *_2_ = 2.7 Hz, 1H), 2.50–2.56 (m, 2H), 2.03–2.22 (m, 2H); ^13^C NMR (D_2_O) *δ* 174.1, 169.7, 77.8, 77.4, 76.9, 55.1, 53.7, 52.6, 30.0, 25.4; ESMS Calculated for C_11_H_13_NO_4_ + Na: 246.0742, Observed 246.0730.

**HCl·H-Thr-OPrp (4h)**

Pale yellow oil; [*α*]_D_  −26 (c = 1, EtOH); FTIR (Neat) 3381 (br), 2128 (w), 1751 (s); ^1^H NMR (D_2_O) *δ* 4.76 (d, *J* = 1.8 Hz, 2H), 4.26–4.33 (m, 1H), 4.01 (d, *J* = 3.6 Hz, 1H), 2.84 (t, *J* = 1.8 Hz, 1H), 1.20 (d, *J* = 6.6 Hz, 3H); ^13^C NMR (D_2_O) *δ* 168.8, 77.8, 77.5, 66.0, 59.2, 55.1, 19.7; ESMS Calculated for C_7_H_11_NO_3_ + Na: 180.0637, Observed 180.0640.

**H-4Aba-OPrp (4i)**

White solid; mp 85°C; FTIR (KBr) 3432 (br), 2125 (w), 1694 (s); ^1^H NMR (CDCl_3_) *δ* 7.88 (d, *J* = 8.7 Hz, 2H), 6.40 (d, *J* = 2.4 Hz, 2H), 4.87 (d, *J* = 2.4 Hz, 2H), 4.11 (bs, 2H), 2.49 (t, *J* = 2.4 Hz, 1H); ^13^C NMR (CDCl_3_) *δ* 151.2, 148.0, 131.9, 118.7, 113.8, 78.2, 74.5, 51.9.; ESMS Calculated for C_10_H_9_NO_2_ + Na: 198.0531, Observed 198.0525.

**H-3Aba-OPrp (4j)**

Pale yellow oil; FTIR (Neat) 3368 (br), 2127 (w), 1716 (s); ^1^H NMR (CDCl_3_ + DMSO-*d_6_*) *δ* 7.35–7.41 (m, 2H), 7.20 (t, *J* = 7.8 Hz, 1H), 6.87–6.90 (m, 1H), 4.88 (d, *J* = 2.7 Hz, 2H), 4.12 (bs, 2H), 2.57 (t, *J* = 2.7 Hz, 1H); ^13^C NMR (CDCl_3_ + DMSO-*d_6_*) *δ* 165.6, 146.7, 129.8, 128.9, 119.4, 118.9, 115.3, 77.4, 74.8, 51.9; ESMS Calculated for C_10_H_9_NO_2_ + H: 176.0711, Observed 176.0709.

### 4.6. Synthesis of Peptides **5a–c**

Boc-amino propargyl esters (**2c, d** and **g**, 2 mmol) were dissolved in 5 mL solution of TFA (50%) in CH_2_Cl_2_. The solution was stirred at rt (28°C) for 1 h and then concentrated under vacuum. The crude TFA salt of the amino propargyl esters were then dissolved in acetonitrile and used for peptide coupling without further purification.

The trifluoroacetate salt of the amino propargyl ester (2 mmol, obtained as above), an *N*-protected amino acid (2 mmol) and HOBt (0·270 g, 2 mmol) were dissolved in acetonitrile (15 mL). The solution was cooled to 0°C and *N*-methylmorpholine (0·24 mL, 2·2 mmol) was added dropwise. A solution of DCC (0·62 g, 3 mmol) in acetonitrile (5 mL) was added to the reaction mixture. Reaction mixture was allowed to come to rt (28°C), and stirring was continued for 4 h. The solvent was removed under vacuum, and the residue was extracted with cold ethyl acetate (50 mL) and filtered over a celite pad. The ethyl acetate solution was washed with saturated citric acid solution (40 mL), saturated Na_2_CO_3_ solution (40 mL), and finally with brine (40 mL). The crude solution of the peptide was dried over anhydrous Na_2_SO_4_ and concentrated. The peptides (**5a**–**c**) were purified by column chromatography (silica gel, 100–200 mesh) eluting with a solution of ethyl acetate (20–40%) in petroleum ether.

**Boc-Ile-Aib-OPrp (5a)**

White solid; mp 117°C; [*α*]_D_  −31 (c = 1, EtOH); FTIR (Neat) 3311 (br), 2133 (w), 1748 (s), 1684 (s), 1652 (s); ^1^H NMR (CDCl_3_) *δ* 6.57 (bs, 1H), 5.08 (bd, *J* = 8 Hz, 1H), 4.71 (d, *J* = 2.7 Hz, 2H), 3.87–3.92 (m, 1H), 2.46 (t, *J* = 2.7 Hz, 1H), 1.85–1.87 (m, 1H), 1.55 (d, *J* = 2.1 Hz, 6H), 1.45 (s, 9H), 1.02–1.21 (m, 1H), 0.89–0.95 (m, 6H); ^13^C NMR (CDCl_3_) *δ* 173.3, 170.8, 155.9, 79.9, 75.0, 59.2, 56.2, 52.8, 37.0, 28.3, 24.7, 15.4, 11.3; ESMS Calculated for C_18_H_30_N_2_O_5_ + Na: 377.2053, Observed 377.2050.

**Boc-Val-Val-OPrp (5b)**

White solid; mp 89°C; [*α*]_D_  −55 (c = 1, EtOH); FTIR (Neat) 3308 (br), 2130 (w), 1749 (s), 1686 (s), 1653 (s); ^1^H NMR (CDCl_3_) *δ* 6.56 (bd, *J* = 8.4 Hz, 1H), 5.18 (bd, *J* = 8.7 Hz, 1H), 4.79 (dd, *J *_1_ = 15.6 Hz, *J *_2_ = 2.4 Hz, 1H), 4.67 (dd, *J *_1_ = 15.6 Hz, *J *_2_ = 2.4 Hz, 1H), 4.56–4.60 (m, 1H), 3.92–3.97 (m, 1H), 2.50 (t, *J* = 2.4 Hz, 1H), 2.08–2.28 (m, 2H), 1.44 (s, 9H), 0.95 (t, *J* = 7.5 Hz, 12H); ^13^C NMR (CDCl_3_) *δ* 171.8, 170.9, 155.8, 79.7, 75.2, 60.0, 56.9, 52.4, 31.1, 30.5, 28.2, 19.2, 18.8, 17.9, 17.6; ESMS Calculated for C_18_H_30_N_2_O_5_ + Na: 377.2053, Observed 377.2055.

**Boc-Phe-Ile-OPrp (5c)**

White solid; mp 109°C; [*α*]_D_  −21 (c = 1, EtOH); FTIR (Neat) 3283 (br), 2136 (w), 1744 (s), 1683 (s), 1658 (s); ^1^H NMR (CDCl_3_) *δ* 7.21–7.32 (m, 5H), 6.40 (bd, *J* = 7.2 Hz, 1H), 5.07 (bs, 1H), 4.72 (dd, *J *_1_ = 15.5 Hz, *J *_2_ = 2.4 Hz, 1H), 4.65 (dd, *J *_1_ = 15.7 Hz, *J *_2_ = 2.4 Hz, 1H), 4.51–4.56 (m, 1H), 4.32–4.39 (m, 1H), 3.07 (d, *J* = 6.9 Hz, 2H), 2.49 (t, *J* = 2.4 Hz, 1H), 1.76–1.89 (m, 1H), 1.33–1.49 (m, 10H), 1.04–1.26 (m, 1H), 0.84–0.91 (m, 6H); ^13^C NMR (CDCl_3_) *δ* 171.0, 170.5, 155.4, 136.6, 129.3, 128.6, 126.9, 80.2, 75.3, 56.4, 55.7, 52.3, 37.9, 28.2, 25.0, 15.2, 11.4; ESMS Calculated for C_23_H_32_N_2_O_5_ + Na: 439.2209, Observed 439.2212.

### 4.7. Synthesis of the Tetrapeptide **9**

The dipeptide **6** was prepared using 3 mmol each of the protected amino acids Boc-Ala-OH (0.57 g) and HCl·H-Phe-OPrp (0.72 g). The peptide coupling was carried out as described for the preparation of **5a**–**c,** and **6** was obtained in 92% yield (1.03 g). The Boc group was deprotected from **6** (0.375 g, 1 mmol) to get **7** using 50% TFA, and the propargyl ester was deprotected from **6** (0.375 g, 1 mmol) to get **8** using tetrathiomolybdate (**1**). The compounds **7** and **8** were coupled using DCC to get the tetrapeptide **9** in 80% yield (0.47 g) after purification using silica gel column chromatography.

**Boc-Ala-Phe-OPrp (6)**

White solid; mp 81°C; [*α*]_D_  −31 (c = 1, EtOH); FTIR (Neat) 3297 (br), 2124 (w), 1752 (s), 1708 (s), 1687 (s); ^1^H NMR (CDCl_3_) *δ* 7.24–7.32 (m, 3H), 7.13–7.16 (m, 2H), 6.59 (bd, *J* = 7.5 Hz, 1H), 4.99 (bs, 1H), 4.85–4.92 (m, 1H), 4.76 (dd, *J *_1_ = 15.5 Hz, *J *_2_ = 2.1 Hz, 1H), 4.67 (dd, *J *_1_ = 15.6 Hz, *J *_2_ = 2.1 Hz, 1H), 4.08–4.17 (m, 1H), 3.19 (dd, *J *_1_ = 14 Hz, *J *_2_ = 6 Hz, 1H), 3.11 (dd, *J *_1_ = 14 Hz, *J *_2_ = 6 Hz, 1H), 2.52 (t, *J* = 2.4 Hz, 1H), 1.43 (s, 9H), 1.31 (d, *J* = 6.9 Hz, 3H); ^13^C NMR (CDCl_3_) *δ* 172.3, 170.5, 155.3, 135.4, 129.4, 128.6, 127.2, 80.1, 76.9, 75.5, 53.0, 52.7, 50.1, 37.6, 28.2, 18.2; ESMS Calculated for C_20_H_26_N_2_O_5_ + Na: 397.1740, Observed 397.1737.

**Boc-Ala-Phe-Ala-Phe-OPrp (9)**

White solid; mp 194°C; [*α*]_D_  −24 (c = 1, EtOH); FTIR (KBr) 3295 (br), 2130 (w), 1750 (s), 1711 (s), 1656 (s); ^1^H NMR (DMSO-*d_6_*) *δ* 8.30 (bd, *J* = 7.2 Hz, 1H), 8.09 (bd, *J* = 7.2 Hz, 1H), 7.67 (bd, *J* = 8 Hz, 1H), 6.99–7.07 (m, 10H), 6.76 (bd, *J* = 7.2 Hz, 1H), 4.48 (s, 2H), 4.27–4.34 (m, 2H), 4.08–4.12 (m, 1H), 3.64–3.71 (m, 1H), 2.77–2.84 (m, 3H), 2.53–2.60 (m, 1H), 1.15 (s, 9H), 0.99 (d, *J* = 6.6 Hz, 3H), 0.86 (d, *J* = 7.2 Hz, 3H); ^13^C NMR (DMSO-*d_6_*) *δ* 172.6, 172.3, 170.51, 170.47, 155.0, 137.6, 136.8, 128.3, 129.1, 128.3, 127.9, 126.6, 126.2, 78.2, 78.0, 77.8, 53.5, 53.4, 52.3, 50.0, 48.0, 37.5, 36.4, 28.1, 18.2, 18.1; ESMS Calculated for C_32_H_40_N_4_O_7_ + Na: 615.2795, Observed 615.2772.

## Figures and Tables

**Table 1:** Preparation and deprotection of propargyl esters.

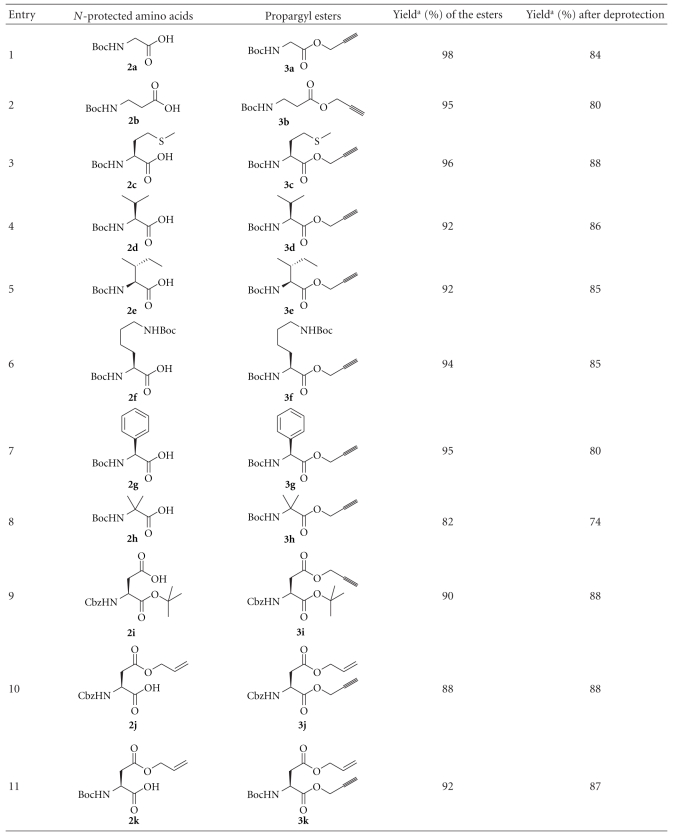 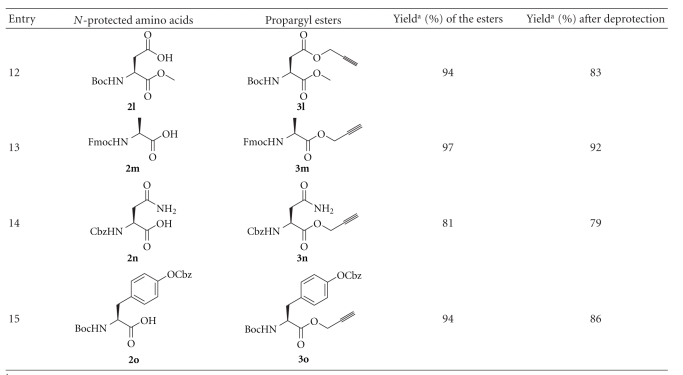

^a^The yields reported are of pure compounds isolated through column chromatography.

**Table 2:** Preparation of propargyl esters of amino acids.

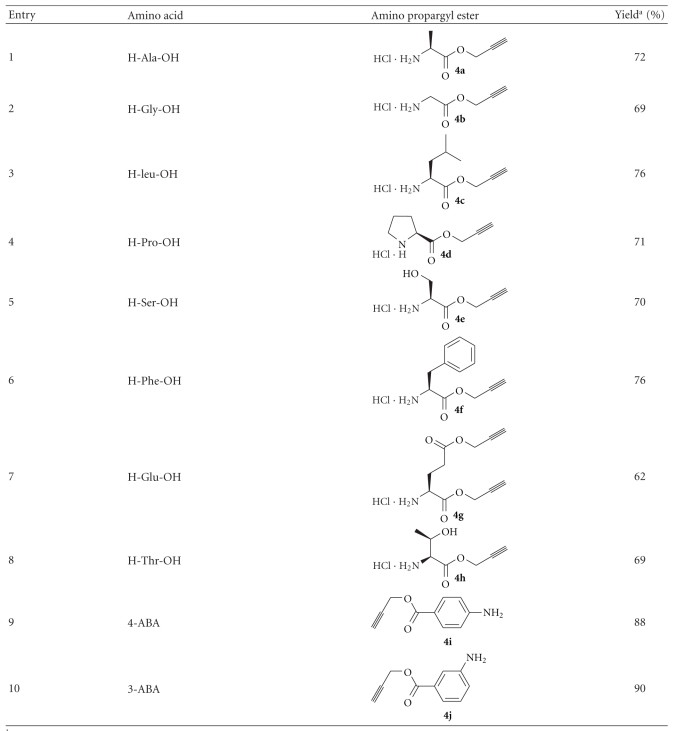

^a^The yields reported are of pure compounds isolated after multiple washings with diethyl ether.

**Table 3:** Preparation of dipeptides from propargyl esters of Aib, Val and Ile.

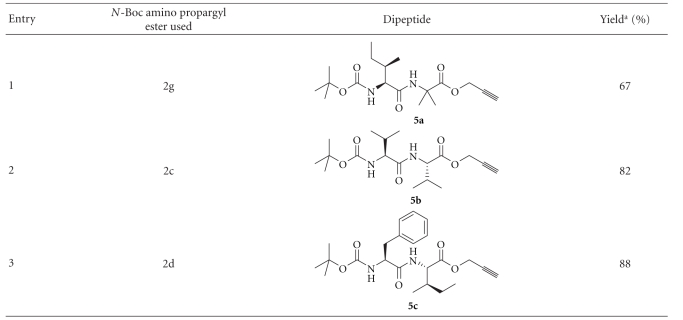

^a^The yields reported are of pure compounds isolated through column chromatography.

## References

[1] Isidro-Llobet A, Álvarez M, Albericio F (2009). Amino acid-protecting groups. *Chemical Reviews*.

[2] Prabhu KR, Devan N, Chandrasekaran S (2002). The Chemistry of tetrathiomolybdate: applications in organic synthesis. *Synlett*.

[3] Swamy VM, Ilankumaran P, Chandrasekaran S (1997). Selective deprotection of propargyl ethers using tetrathiomolybdate. *Synlett*.

[4] Ilankumaran P, Manoj N, Chandrasekaran S (1996). Prop-2-ynyl as a protective group for carboxylic acids: a mild method for the highly selective deprotection of prop-2-ynyl esters using tetrathiomolybdate. *Chemical Communications*.

[5] Sinha S, Ilankumaran P, Chandrasekaran S (1999). The prop-2-ynyloxy carbonyl function (POC): a new amino-protecting group removable from sulfur-containing peptides by ultrasonic irradiation with tetrathiomolybdate under mild and neutral conditions. *Tetrahedron Letters*.

[6] Bhat RG, Sinha S, Chandrasekaran S (2002). Propargyloxycarbonyl (Poc) amino acid chlorides as efficient coupling reagents for the synthesis of 100% diastereopure peptides and resin bound tetrathiomolybdate as an effective deblocking agent for the Poc group. *Chemical Communications*.

[7] Sridhar PR, Chandrasekaran S (2002). Propargyloxycarbonyl (Poc) as a protective group for the hydroxyl function in carbohydrate synthesis. *Organic Letters*.

[8] Ramesh R, Bhat RG, Chandrasekaran S (2005). Highly selective deblocking of propargyl carbonates in the presence of propargyl carbamates with tetrathiomolybdate. *Journal of Organic Chemistry*.

[9] Ramesh R, De K, Gupta S, Chandrasekaran S (2008). Propargyloxycarbonyl as a protecting group for the side chains of serine, threonine and tyrosine. *Journal of Chemical Sciences*.

[10] Kim S, Lee JI, Kim YC (1985). A simple and mild esterification method for carboxylic acids using mixed carboxylic-carbonic anhydrides. *Journal of Organic Chemistry*.

